# Recent trends of non-communicable diseases in Cambodia: a narrative review of challenges, risk factors, and public health strategies

**DOI:** 10.3389/fpubh.2026.1799131

**Published:** 2026-03-13

**Authors:** Virak Sorn, Monirath Suon, Sin Chea

**Affiliations:** 1 Faculty of Health Sciences and Biotechnology, University of Puthisastra, Phnom Penh, Cambodia; 2 Faculty of Nursing and Midwifery, University of Puthisastra, Phnom Penh, Cambodia; 3 University of Puthisastra, Phnom Penh, Cambodia

**Keywords:** Cambodia, epidemiological transition, low- and middle-income countries, non-communicable diseases, public health policy

## Abstract

Non-communicable diseases (NCDs) pose a major public health issue in low- and middle-income countries (LMICs), with Cambodia facing a substantial and increasing burden. This narrative review complies with national data and policy-relevant findings to address the epidemiology of significant NCD categories, identify underlying risk factors, and evaluate systematic challenges to provide effective prevention and care. In addition, it also assesses current national strategies and highlights the most important areas for intervention, including primary prevention, expanded screening, sustainable health financing and intersectoral action. Addressing the increase of NCDs requires coordinated effort among governments, healthcare providers, and communities through comprehensive intersectional strategies. Lessons from the Cambodian experience are intended for future research, public policy, and NCD interventions in similar LMICs that are undergoing rapid demographic and epidemiological transitions, thereby promoting more effective and equitable NCD control.

## 1 Introduction

Globally, NCDs have emerged as the leading cause of morbidity and mortality, accounting for approximately 73% of all deaths worldwide in 2021, with the greatest burden of concern in LMICs (1). This epidemiological transition presents considerable challenges for healthcare systems that are tasked with addressing a dual burden comprising enduring communicable diseases (CDs) alongside the growing demand for long-term NCD prevention and management. The increasing burden of NCDs imposes significant economic costs through reduced labor productivity and increased healthcare expenditure, with implications for national development. Without effective intervention, the NCD burden is expected to continue rising, particularly in LMICs, where capacity for early detection, continuous care, and long-term disease management remains limited (2). In response, the World Health Organization (WHO) established the Global Action Plan for the Prevention and Control of NCDs, setting targets for 2025 and 2030 and emphasizing a comprehensive, multi-sectoral approach integrates population-level prevention, health system strengthening, and policy interventions to reduce premature NCD-attributable mortality and disability (3).

Within Southeast Asia, the rise of NCD reflects not only successful CD control and increased life expectancy but also broader social, economic, and nutritional transitions associated with urbanization and globalization (4–6). Sedentary lifestyle, unhealthy dietary pattern, tobacco use, and harmful alcohol consumption have become increasingly prevalent, particularly among urban and socioeconomically vulnerable populations (7, 8). Conditions such as obesity and diabetes have gained increasing attention as manifestations of these transitions, underscoring the evolving and complex interplay between CDs and NCDs dynamics across the region (9).

Cambodia exemplifies these challenges as an LMIC undergoing rapid epidemiological and demographic transition. Despite notable progress in controlling infectious disease, the country faces a sharply rising NCD burden with significant implications for population health system capacity and national development (7, 8). Modifiable behavioral risk factors, dietary shifts toward energy-dense foods, and urbanization-driven change to physical environments have collectively amplified NCDs risk across the population (10–12). Healthcare gaps, particularly in underserved rural communities, further constrain the capacity for early detection, continuous care, and long-term disease management, leaving a substantial proportion of the population without adequate NCD services (7).

Understanding the full scope of Cambodia’s NCD burden, its underlying determinants, and the adequacy of current prevention and control responses is therefore essential to inform effective and equitable public health policy. This study aimed to examine the burden of NCDs in Cambodia by describing the epidemiological and mortality patterns of major NCD categories, identifying key risk factors and urban–rural disparities, and assessing gaps in healthcare access and quality of care. This further sought to evaluate existing national strategies and policy frameworks for NCD prevention, screening, and management, while drawing transferable lessons for comparable LMIC settings undergoing rapid demographic and epidemiological transition, ultimately informing future research and public health policy to support more effective and equitable NCD responses.

This article aimed to examine the burden of NCDs in Cambodia by describing the epidemiological and mortality patterns of major NCD categories, identifying key risk factors and urban–rural disparities, and assessing gaps in healthcare access and quality of care. This further sought to evaluate existing national strategies and policy frameworks for NCD prevention, screening, and management, while drawing transferable lessons for comparable LMIC settings undergoing rapid demographic and epidemiological transition, ultimately informing future research and public health policy to support more effective and equitable NCD responses.

## 2 Methods

This study utilized narrative review methodology to synthesize existing evidence on the burden, risk factor, healthcare challenge, and strategic response related to NCDs in Cambodia with broader literature on comparable LMIC settings.

### 2.1 Literature search and source selection

A comprehensive literature search was conducted across multiple sources, including peer-reviewed journals, global health organization reports, and grey reports. Those sources included World Health Organization, Ministry of Health of Cambodia, International Diabetes Federation, World Bank, United Nations Development Program (UNDP), and other international and regional organizations. The literature spanned from 2017 to 2025 with a deliberate emphasis on literature published between 2020 and 2025 to capture the most current epidemiology and policy evidence.

### 2.2 Eligible criteria

Literature was selected based on relevance to the following areas: (1) epidemiology and burden of NCDs globally, regionally, and specifically in Cambodia; (2) major NCD categories, including cardiovascular disease, cancer, chronic respiratory disease, diabetes, and mental health conditions; (3) modifiable risk factors, such as obesity, dietary pattern, physical inactivity, tobacco use, and alcohol consumption; (4) healthcare system capacity, access, and quality of NCD in Cambodia and comparable LMICs; (5) prevention, screening, early detection, and management strategies; (6) health financing, universal health coverage, and intersectional policy response.

## 3 Non-communicable diseases overview

NCDs constitute a substantial and growing share of the global disease burden. In addition to their independent contribution to morbidity and mortality, certain infectious diseases and early-life exposures have been shown to increase the risk of developing specific NCDs later in life, underscoring the interconnected nature of disease pathways across the life course (13). NCDs are typically chronic in nature and arise from a complex interaction of genetic susceptibility, physiological processes, environmental exposures, and behavioral factors (1). The most common NCDs include cardiovascular diseases (e.g., heart attack and stroke), cancers, chronic respiratory diseases (e.g., chronic obstructive pulmonary disease and asthma), diabetes, mental health conditions, and neurological disorders (14). NCD risk factors affect individuals across the life span, with children, adults, and older populations exposed to behavioral and environmental risks such as unhealthy diets, physical inactivity, tobacco use, harmful alcohol consumption, and air pollution (1). Beyond their direct health effects, NCDs impose significant psychosocial burdens on patients and caregivers, often leading to reduced quality of life and long-term economic consequences (15).

### 3.1 Global burden of NCDs

NCDs remain the leading cause of death and disability worldwide. In 2021, they accounted for approximately 43–44 million deaths globally and an estimated 1.7 billion disability-adjusted life years (DALYs), reflecting both premature mortality and prolonged disability (16). Cardiovascular diseases, cancers, chronic respiratory diseases, and diabetes collectively account for the majority of this burden (17). Although age-standardized mortality rates for some NCDs have declined in several high-income countries, the absolute burden continues to rise globally due to population growth and aging.

The distribution of the NCD burden is highly unequal. Countries with lower socio-demographic index (SDI) levels experience disproportionately higher rates of premature NCD mortality, reflecting constrained health system capacity, delayed diagnosis, and limited access to long-term care (18). Of the estimated NCD-related deaths occurring before the age of 70 years, more than four-fifths are concentrated in LMICs (1). Major modifiable risk factors contributing to the global NCD burden include elevated blood pressure, unhealthy diets, tobacco use, and harmful alcohol consumption, which together account for a large share of preventable DALYs (16). These patterns highlight the urgency of targeted prevention and health system strengthening in resource-constrained settings.

### 3.2 Burden of NCDs in Southeast Asia

Southeast Asia is experiencing a rapid epidemiological transition, with NCDs now accounting for 55% of all deaths in the region—an estimated 7.9 million annually (19). Cardiovascular diseases and cancers are the leading contributors, while chronic respiratory diseases and diabetes continue to rise in prevalence. Neurological conditions, particularly stroke and dementia, are emerging as significant contributors to disability (an estimated 64.4 million DALYs in the region), reflecting population aging and persistent vascular risk factors (20, 21).

Diabetes represents a particularly pressing concern. By 2050, it is projected that 184.5 million individuals in Southeast Asia will be living with diabetes, marking a 36% increase to an estimated prevalence of 13.2% (22). Notably, the region has the third-highest global rate of undiagnosed diabetes, with 42.7% of cases remaining unidentified (22). A large proportion of individuals with diabetes remain unaware of their condition, delaying treatment initiation and increasing the risk of complications. While demographic aging contributes to these trends, broader structural factors—including urbanization, changing dietary patterns, and reduced physical activity—play a critical role (5, 6). Other significant NCD burdens include injuries, mental health disorders, and conditions affecting the eyes, oral cavity, and ears (17, 20–22). These dynamics are increasingly affecting younger adult populations, with important implications for workforce productivity and long-term health system demand, raising further public health concerns (23).

### 3.3 Burden of NCDs in Cambodia

In Cambodia, NCDs have become a dominant public health challenge, accounting for approximately two-thirds of all deaths—an estimated 60,000 annually—according to recent national and international estimates (24). Cardiovascular diseases, particularly hypertension and stroke, represent the largest share of NCD-related mortality and morbidity and are closely linked to dietary change, physical inactivity, and tobacco use (25). Diabetes prevalence is also increasing. The International Diabetes Federation estimates that more than 720,000 adults aged 20–79 years were living with diabetes in Cambodia in 2024, with numbers projected to rise substantially to 1.2 million by 2050—a 66% increase if current trends persist (22).

Cancer constitutes another major component of Cambodia’s NCD burden. The most commonly reported cancers include liver, lung, breast, colorectal, and cervical cancers, reflecting a combination of infectious, behavioral, and environmental risk factors (26). Lung cancer is the leading cause, with 2.5 million new cases and 1.8 million associated deaths, representing 18.7% of total cancer-related mortality globally (27). Chronic respiratory diseases, including asthma and chronic obstructive pulmonary disease, further contribute to morbidity, driven in part by air pollution and high rates of tobacco exposure (28). While national cancer registry and surveillance data remain limited, available evidence indicates a growing demand for diagnostic and treatment services, placing additional strain on the health system. Addressing these interconnected health issues through improved prevention, management, and healthcare access is crucial for reducing the impact of NCDs in Cambodia (29, 30).

Modifiable lifestyle and metabolic risk factors play a central role in shaping Cambodia’s NCD profile. Among women of reproductive age, 33% reported consuming unhealthy diets, including high levels of sugary beverages and alcohol—key contributors to NCD risk (31). Recent national data indicate rising levels of overweight (22.56%) and obesity (5.61%) among adult women, with higher prevalence observed among older age groups, married individuals, and urban residents (32). In specific subpopulations, such as adults living with HIV, studies have documented a notable prevalence of cardiometabolic conditions—including hypertension (8.8%), diabetes (15.1%), and dyslipidemia (34.7%)—many of which remain undiagnosed prior to screening (33). These findings underscore persistent gaps in early detection and highlight the importance of integrating NCD screening and prevention into routine health services across the country (Table 1).

**Table 1:** Comparative burden of NCDs globally, in Southeast Asia, and in Cambodia (2000–20) (1, 7, 23, 24, 29, 34, 35).

Year	Global (% of all deaths due to NCDs)	Southeast Asia (% of deaths due to NCDs)	Cambodia (% of deaths due to NCDs)
2000	60%	50%	46%
2010	63%	55%	54%
2015	70%	62%	60%
2020	74%	69%	64%

## 4 Epidemiology of NCDs in Cambodia

Non-communicable diseases (NCDs) account for approximately 64% of all deaths in Cambodia, reflecting a substantial and growing public health burden (29). This rising dominance of NCDs places increasing strain on a health system historically oriented toward communicable disease control and maternal and child health. The epidemiological profile of NCDs in Cambodia highlights significant challenges associated with increasing prevalence, sustained premature mortality, population aging, and rapid urbanization, all of which complicate the organization and delivery of healthcare services (34, 36).

The NCD burden is not evenly distributed across the population. Certain demographic groups—including women of reproductive age and people living with HIV—appear to be disproportionately affected by metabolic and behavioral risk factors (37). Recent studies document concerning patterns of unhealthy behaviors and associated health outcomes, underscoring the need for strengthened prevention and early intervention strategies (38). National survey data indicate that approximately one-third of women aged 15–49 years report unhealthy dietary practices, and more than half consume sugar-sweetened beverages, both of which are established contributors to cardiometabolic risk (32). Over the past two decades, the prevalence of overweight and obesity among women of reproductive age has increased substantially, rising from approximately 6% in 2000 to more than 20% in recent surveys, with higher risk observed among older, married women and those from wealthier households (33).

According to a recent STEPS survey, over 20% of individuals are overweight, 17% have raised blood pressure, and 6.3% exhibit abnormal blood glucose levels. Moreover, average daily salt consumption is estimated to be nearly twice the World Health Organization’s recommended maximum, indicating widespread exposure to dietary risk factors (24). Despite this burden, screening coverage remains limited: more than 40% of adults report never having had their blood pressure measured, and over three-quarters have never been screened for diabetes, highlighting substantial gaps in early detection and preventive care (39).

### 4.1 Prevalence rates

NCDs are the leading cause of death worldwide, and their prevalence continues to increase in many LMICs, including Cambodia. This trend underscores the importance of robust national surveillance systems aligned with the Sustainable Development Goals (SDGs) and WHO monitoring frameworks (4, 34). In Cambodia, the most prevalent NCDs are cardiovascular diseases, cancers, chronic respiratory diseases, and diabetes, which together account for the majority of NCD-related morbidity and mortality. Other chronic conditions, such as mental and neurological disorders and chronic kidney disease, contribute to overall disease burden but are less well characterized due to data limitations.

The rising prevalence of these conditions is closely linked to structural and behavioral changes, including urbanization, shifts toward more sedentary lifestyles, increased availability of energy-dense processed foods, and ongoing exposure to tobacco and alcohol (1, 7). These drivers reflect broader social and economic transitions rather than isolated individual behaviors, highlighting the need for population-level prevention strategies alongside clinical care.

### 4.2 Trends over time

Cambodia has experienced a marked epidemiological transition over the past decade, with the proportion of deaths attributable to NCDs increasing from approximately 54% in 2010 to 64% in recent estimates (7, 29). Available national surveys and modeled estimates suggest upward trends in several major NCD categories, including cardiovascular disease mortality, which has increased by 42%; cancer-related deaths, by 65%; and diabetes-related deaths, by 90%, although the magnitude of change varies across data sources and indicators (34).

Metabolic risk factors have also increased over time. Successive STEPS surveys indicate a rising prevalence of hypertension from 18 to 30%, while diabetes prevalence doubled from 5 to 10% between 2010 and 2023 (22). Obesity prevalence has increased notably since the late 2000s, particularly in urban areas, reflecting changes in diet and physical activity patterns. While processed food consumption and reduced physical activity are frequently cited as contributing factors, precise quantification of these drivers remains limited by data availability. The obesity epidemic has also intensified, affecting 16% of adults in 2023 compared to just 5% in 2008. This trend is largely attributed to a 300% increase in processed food consumption since 2010 (34).

Some progress has been observed in specific areas of risk reduction. For example, national data indicate a decline in tobacco use from 32 to 22% between 2014 and 2023, following the implementation of stricter tobacco control policies, suggesting that regulatory interventions can yield measurable population-level benefits (40). Nevertheless, the overall trajectory of NCD burden remains upward. Economic analyses suggest that, without expanded prevention, early detection, and effective long-term management, NCD-related healthcare costs and productivity losses could place a substantial burden on Cambodia’s economy, up to 5% of Cambodia’s GDP by 2030 (29). These trends underscore the urgency of strengthening comprehensive, equitable, and sustainable NCD responses.

## 5 Major non-communicable diseases in Cambodia

NCDs constitute the dominant cause of mortality in Cambodia, accounting for approximately two-thirds of all deaths (29). Cardiovascular diseases, cancers, chronic respiratory diseases, and diabetes together represent the largest contributors to NCD-related morbidity and mortality, consistent with global and regional patterns observed in LMICs (7). Mental health conditions and chronic kidney disease also contribute to the national disease burden, although these conditions are less comprehensively captured in routine surveillance systems.

These major NCDs share a set of modifiable behavioral and environmental risk factors, including tobacco use, harmful alcohol consumption, unhealthy diets, physical inactivity, and exposure to indoor and ambient air pollution. Recent national survey data indicate persistently high levels of tobacco exposure, particularly among men (42.5% of adult males in Cambodia smoke cigarettes, and 2 million people use tobacco products), alongside widespread dietary inadequacy (80%) characterized by low fruit and vegetable intake and excessive consumption of salt and saturated fats, contributing to 20% of adults having high cholesterol and 10% suffering from hypertension (34). These risk profiles contribute to a substantial prevalence of metabolic conditions, including hypertension, dyslipidemia, and diabetes, which remain underdiagnosed and suboptimally managed in many settings due to barriers in access to care and continuity of treatment (22).

Cancer represents a growing area of concern, with liver, lung, breast, colorectal, and cervical cancers among the most commonly reported types. These patterns reflect the combined influence of infectious agents—such as hepatitis B virus and human papillomavirus—as well as tobacco use and other lifestyle-related risk factors (34). Cervical cancer is the leading malignancy among women, with an incidence rate of 13.5 per 100,000 and a mortality rate of 10.1 per 100,000 (41). Chronic kidney disease is increasingly recognized as a downstream consequence of poorly controlled hypertension and diabetes, compounded in some cases by the use of nephrotoxic traditional medicines (42). Despite policy efforts to strengthen NCD prevention and control, challenges related to early diagnosis, treatment adherence, and equitable service coverage persist across disease categories.

### 5.1 Cardiovascular diseases

Cardiovascular diseases (CVDs) are a leading public health concern in Cambodia, with hypertension serving as a key risk factor. The prevalence of hypertension among adults stands at approximately 35.2%, with strong associations found between CVDs, obesity, and diabetes (43). Obesity affects 15% of the adult population (7, 27). The situation has deteriorated in recent years. According to the Ministry of Health’s 2025 National Health Report, hospitalizations related to CVDs increased by 29.53%, while the number of hypertension patients treated in public facilities rose by 21.44% compared to 2022–23. Alarmingly, new hypertension cases surged to 330,985 in 2024—up from 295,461 in 2023—signaling a rapidly escalating burden (44). In 2024, a total of 13,716 patients sought treatment for cardiovascular conditions: heart failure (8,087 cases), angina (1,898), acute myocardial infarction (388), stroke (1,025), and stroke-related complications (2,318). Hospital admissions totalled 22,218, with stroke (8,174), heart failure (5,214), and angina (4,129) comprising the majority of cases. CVDs claimed 1,074 lives in 2024, with heart failure (347 deaths) and stroke (271 deaths) being the leading causes (44).

### 5.2 Cancer

Cancer poses a growing public health challenge in Cambodia. In 2024, the country recorded 19,795 new cancer cases and 13,799 deaths among a population of 17.2 million (26). Liver cancer was the most prevalent (17.7% of all cases, 23.8% of cancer deaths), followed by lung cancer (10.9% of cases, 13.9% of deaths) and breast cancer (10.7% of cases, 6.6% of deaths). Gender disparities were evident: liver, lung, and colorectal cancers were most common in men, while breast, cervical, and liver cancers predominated among women. Recent data from the Ministry of Health’s National Health Report indicate a substantial rise in cancer treatment cases—from 16,817 in 2023 to 31,518 in 2024. Women made up 61% of all patients receiving treatment. The most frequently hospitalized cancers included breast (20.4%), cervical (11.5%), colon (7.7%), liver (7%), lung (6%), ovarian (4.1%), rectal (3.2%), stomach (3%), and oral cancers (2.1%), while other cancers made up 35% of hospitalizations (44).

### 5.3 Chronic respiratory diseases

Chronic respiratory diseases (CRDs) remain a pressing NCD concern in Cambodia, although incomplete surveillance systems hinder accurate estimates of their incidence and prevalence. High rates of stunting and undernutrition in children, combined with maternal exposure to biomass fuels and increased indoor air pollution, suggest a growing CRD burden—particularly in urban settings. Cambodia has incorporated CRD prevention and control into national health development strategies, recognizing their potential long-term impact (34, 44).

However, tuberculosis (TB) and acute respiratory infections (ARI) remain persistent public health threats. A study on patients presenting with respiratory symptoms found that 30% tested positive for TB, 44% for other bacterial infections, and 9% had co-infections, highlighting the overlap between infectious and chronic respiratory conditions (45). While ARI symptoms among children under five have decreased since 2,000, the burden remains high. Risk factors include young age, maternal smoking, inadequate hygiene, and exposure to indoor pollutants (46). CRDs in Cambodia require continued attention, particularly in light of overlapping infectious disease patterns and increasing urban environmental risks.

### 5.4 Diabetes

Diabetes is a chronic metabolic condition caused by insufficient insulin production or inefficient insulin utilization in our body, leading to elevated blood glucose levels. In Cambodia, both type 1 and type 2 diabetes are becoming increasingly prevalent. In 2024, newly diagnosed diabetes cases rose sharply to 230,279 (17,166 cases of type 1 diabetes and 213,113 cases of type 2 diabetes), compared to 144,668 in the previous year (16,304 type 1 and 128,364 type 2) (44). Hospitalizations for diabetes also increased—from 12,238 in 2023 to 17,534 in 2024. Of these, 15,797 cases were attributed to type 2 diabetes. This trend reflects not only the growing burden of diabetes in Cambodia but also gaps in prevention, early detection, and treatment adherence.

## 6 Risk factors for NCDs in Cambodia

NCDs are a significant global public health concern, including in Cambodia. Risk factors refer to elements that increase the likelihood of developing or worsening a disease or health condition (47). These are broadly categorized into modifiable and non-modifiable risk factors. Non-modifiable risk factors relate primarily to age and genetic predisposition (48), whereas modifiable risk factors include tobacco use, obesity, poor diet, harmful alcohol consumption, and physical inactivity. Globally recognized risk factors also encompass dietary habits, high body mass index (BMI), elevated fasting plasma glucose, impaired glomerular filtration rate, exposure to secondhand smoke, sociodemographic index, and alcohol use (47). Modifiable risk factors are the principal contributors to increased morbidity and mortality from NCDs, although age and family history remain important non-modifiable factors influencing disease classification. In Cambodia, diabetes, cardiovascular diseases, and cancer are among the most prevalent NCDs, accounting for a high proportion of premature deaths. This section outlines three main categories of risk factors—behavioral, environmental, and metabolic—that are critical in shaping the country’s NCD landscape.

### 6.1 Behavioral risk factors

Behavioral risk factors relate to the choices and habits individuals adopt in their daily lives, regardless of whether these are directly linked to a specific medical condition. Key behavioral risk factors for NCDs include tobacco use, excessive alcohol intake, poor dietary practices, and physical inactivity. These are widespread in Cambodia and contribute significantly to NCD-related mortality (49). Although behavioral change is inherently challenging, the burden of NCDs will continue to rise unless these risk factors are effectively identified and addressed through appropriate prevention strategies. Furthermore, the economic burden of unmanaged behavioral risk factors may be substantial for a country with limited health resources (47). While much of the current epidemiological research focuses on the prevalence and impact of NCDs, there is comparatively little attention paid to the underlying behavioral drivers. Therefore, the discussion of common risk factors and behavioral risk factor interventions has received special attention in this piece of paper.

### 6.2 Environmental risk factors

Environmental risk factors represent some of the most significant contributors to the NCD burden (50). These risks are often linked to unfavorable living and working conditions, such as exposure to chemical, biological, physical, or neurotoxic agents that may have immediate or long-term impacts on health (51). In Cambodia, while the agrarian economy and relatively stable infrastructure offer a level of resilience, poor environmental management—especially in relation to natural resources and pollution—poses serious and growing health threats if not addressed with effective safeguards.

### 6.3 Metabolic risk factors

Metabolic risk factors are key determinants of NCDs and include obesity, hypertension, dyslipidemia, and diabetes. Atherogenic lipid profiles, together with abnormal glucose metabolism and elevated blood pressure, are increasingly used to assess cardiometabolic risk in adults (52). These conditions, whether present individually or in combination (i.e., metabolic syndrome), are major contributors to kidney failure, cardiovascular events, and broader NCD-related mortality.

The socio-economic, healthcare, and quality-of-life impacts of metabolic risk factors are immense and escalating. Addressing these risks demands action not only through national public health strategies but also at the level of individual behavior change and community health education. Cambodia, as part of the global health community, has responded by formulating and initiating strategic action plans to prevent and control NCDs (7, 34). However, current strategies have not yet yielded the desired outcomes in terms of curbing metabolic risk factors. Although NCD-related health services are designed to deliver structured and quality care alongside health promotion efforts, more comprehensive and inclusive approaches are required—particularly those that target all age groups through preventive care and population-wide health initiatives (37). Since there is limited locally generated data, this paper provides a concise and evolving overview of the prevalence, risk factors, and potential prevention strategies associated with NCDs in Cambodia. Future research should seek to generate context-specific, evidence-based interventions, with an emphasis on public health interventions that are both culturally appropriate and outcome-oriented.

## 7 Challenges in addressing NCDs in Cambodia

Currently, Cambodia is contending with a double burden of disease: both CDs and NCDs. The high prevalence of major NCDs and their associated risk factors presents significant challenges, including limited awareness of these risk factors, a general underestimation of the NCD burden, and insufficient healthcare resources (29, 37). Evidence suggests that the high rates of NCDs are closely linked to the increasing prevalence of chronic kidney disease. Modifiable risk factors—such as alcohol consumption and irregular fruit intake—alongside non-modifiable ones like advanced age and a family history of diabetes—contribute to the country’s growing diabetes burden (34). Although NCDs are a major health concern in Cambodia, underdiagnosis remains widespread, meaning actual prevalence rates are likely far higher than reported. Key modifiable risk factors include high salt intake, low consumption of fruits and vegetables, and harmful patterns of alcohol use (7). Addressing these growing challenges requires a multifaceted approach, including improvements in healthcare infrastructure, health education, workforce development, and financial investment.

### 7.1 Healthcare infrastructure

Cambodia’s healthcare system is pluralistic, comprising both a large private sector and a public healthcare network. The public health system operates on three levels: national, provincial, and operational district (OD). At the central level are national hospitals, specialized centers, training institutions, and various departments under the Ministry of Health. The provincial level includes 25 provincial health departments that serve as intermediaries between the central and district levels. The OD level—comprising 92 referral hospitals, 1,222 health centers, and 128 community health posts—functions as the primary healthcare delivery system and is the closest level to local communities (37).

Since 2019, the Cambodian government has decentralized certain responsibilities, transferring the management of health services, human resources, and financing to provincial administrations. The central government now focuses on policymaking and regulatory oversight, while provinces oversee service delivery. Typically, each OD serves between 100,000 and 200,000 residents and contains at least one referral hospital (53). The country’s development has been impacted by armed struggles and under-resourced civil services. The government has made progress in reforming civil services since the last three decades, aiming to improve human resources, operational efficiencies, and structural organization (34). Despite structural reforms and decades of development, the healthcare system still faces challenges, particularly in rural areas, due to a lack of medical personnel, equipment, supplies, and difficult geographical terrain. These operational constraints severely impact the overall effectiveness of Cambodia’s health infrastructure.

### 7.2 Health education and awareness

Raising awareness and improving knowledge about NCDs, their risk factors, complications, and prevention strategies is vital to controlling their spread (47). Studies have shown that awareness levels vary across different sociodemographic groups and are often linked to health-seeking behavior. A lack of understanding contributes to delayed diagnosis and treatment.

Health education initiatives should aim to raise awareness of modifiable risk factors and symptoms through screening and information campaigns (54). Primary prevention—often more cost-effective than secondary or tertiary care—should be a central pillar of national NCD strategies. It should focus on lifestyle modifications, behavior change, and reducing exposure to risk factors (47). Prevention strategies must begin at the community level. These include school-based education, workplace wellness programs, and community facilities promoting physical activity and healthy diets. Campaigns using mass media and social media can also reinforce these messages (7). Evidence suggests that even a modest improvement in health awareness can lead to a 4% reduction in the prevalence of hypertension, type 2 diabetes, dyslipidemia, and obesity (12). Health education should begin early in life to discourage the adoption of unhealthy behaviors. Interventions targeting high school students are particularly effective in shaping long-term habits. Moreover, the right to health information is recognized in multiple international declarations as a fundamental human right. Numerous studies have shown that better awareness and health knowledge significantly improve early diagnosis and disease prevention outcomes (55, 56). Lower health knowledge is associated with higher blood pressure, poor diet, and poor healthy lifestyle practices.

### 7.3 Healthcare workforce

Cambodia faces a significant shortage of healthcare professionals, including doctors, nurses, and specialists. The country has only 1.4 doctors per 1,000 people, well below the World Health Organization’s recommended threshold. While this figure improved slightly to 2.0 per 1,000 in 2023, it still places Cambodia near the bottom among ASEAN countries. The government aims to raise this ratio to 2.4 by 2030 (57). Retention of healthcare professionals is especially problematic in rural areas, where working conditions and compensation are often suboptimal. Many trained professionals migrate to urban areas or join the private sector. This workforce shortage results in longer waiting times, overburdened staff, and diminished quality of care—particularly for patients with chronic NCDs (30).

### 7.4 Healthcare financing

Inadequate financial investment remains one of the main barriers to improving healthcare in Cambodia. Limited funding affects the availability of essential medicines, diagnostic tools, and facility maintenance. Financial constraints are particularly problematic for NCD-specific services, including screening, early detection, and long-term management (29). Moreover, the integration of NCD services into primary healthcare remains limited, resulting in fragmented care and inefficiencies. Out-of-pocket expenses (OOPE) in Cambodia still remain high (61.21%) in 2022 compared to neighboring countries like Vietnam (39.55%) and Thailand (9.21%) (58), while OOPE for diagnostics, medication, and long-term treatment are often unaffordable for many Cambodians, especially those in rural or marginalized communities (37). Heavy reliance on external donors has rendered NCD programs unsustainable in the long term. Without increased domestic financing, it will be difficult to ensure consistent and stable, high-quality services—especially in areas where budget limitations are most acute (7).

## 8 Prevention and control strategies of NCDs in Cambodia

In Cambodia, the prevalence of NCDs has significantly increased, posing a major public health concern nationwide. The most common NCDs include cardiovascular diseases, cancers, chronic respiratory diseases, and diabetes, primarily driven by behavioral and dietary risk factors such as unhealthy eating habits, sedentary lifestyles, alcohol consumption, and tobacco use (1). In recent years, the Cambodian government has initiated efforts to combat NCDs by promoting physical activity and healthier dietary practices (7). Although progress has been made—particularly in tobacco control through advertising restrictions and the development of NCD policies and targets—significant gaps remain in addressing other major risk factors. Areas such as national NCD guidelines, tobacco metrics, and alcohol-related indicators lack adequate policy implementation and enforcement (59). Consequently, Cambodia still faces a long road toward achieving its NCD targets.

From a health systems perspective, Cambodia must adopt a series of NCD control measures in the coming years. Historically, the country prioritized curative care in response to both CDs and early-stage NCDs. Over the last two decades, however, the health system has undergone marked improvements, especially in maternal and child health and the incorporation of preventative care. Immunization campaigns and maternal care programs contributed to a 54% decline in neonatal and under-five mortality rates between 2014 and 2022—surpassing global averages—and increased skilled birth attendance to 98.7% (60). In parallel, Cambodia has adopted a proactive approach to NCDs control by developing strategic plans and national reports (61). The country now embraces a multifaceted framework that emphasizes both strengthening the health system and reducing individual risk factors.

### 8.1 Primary preventive strategies

A healthy lifestyle is fundamental to the primary prevention of NCDs. These diseases are influenced by both modifiable and non-modifiable risk factors, with the former offering key opportunities for intervention. Physical inactivity, poor dietary choices, tobacco use, excessive alcohol consumption, hypertension, hypercholesterolemia, overweight, and obesity are among the leading risk factors that can be mitigated through primary prevention efforts (62). Individual behavior and lifestyle significantly influence the onset of NCDs, making behavior modification a central aim of public health strategies (63). Effective interventions include smoking cessation, moderate alcohol intake, a balanced diet, and regular physical activity (64). Now is a crucial moment to act and prevent future health crises.

Targeted prevention programs can address varying levels of disease susceptibility, even in cases where NCDs or their complications have already emerged. Since lifestyle changes tackle the root causes of many NCDs—especially dietary habits, sedentary behavior, and tobacco exposure—they remain the most cost-effective and impactful preventive measures (65). Nonetheless, functional, structural, and financial barriers continue to hinder the development and execution of effective prevention policies. A comprehensive prevention approach should prioritize reducing tobacco and alcohol use, promoting physical activity, and encouraging healthy eating habits.

### 8.2 Secondary preventive strategies

Secondary prevention focuses on detecting diseases early and managing them effectively to prevent complications. In Cambodia, where access to essential diagnostics remains limited, regular screening is critical for early diagnosis and timely intervention. Promoting self-examination and routine health checks can significantly improve treatment outcomes for NCDs (66). Many complications can be addressed without specialist intervention, provided that patients are regularly monitored, treatment regimens are updated, and patient education is prioritized. Chronic conditions such as diabetes require consistent screening and management. For instance, the Brazilian Society of Diabetes recommends the measurement of HbA1c to monitor blood glucose levels in individuals with type 2 diabetes (67). The primary aim of secondary prevention is to reduce health risks, minimize disability and distress associated with chronic illness, and extend healthy life expectancy. Despite its importance, secondary prevention has received limited attention in Cambodia. Greater focus on this level of intervention is essential and warrants further research and policy development.

Yet, community-based approaches like school nutrition programs, village health volunteers or support groups, and mobile health (mHealth), such as short message services (SMS) campaigns, have demonstrated potential in reaching marginalized communities and may be the best course of action. Using SMS reminder strategies has been shown to have a positive impact in multiple studies globally, with the percentage of people who enrolled in hypertension screening increasing between 5 and 10% after receiving an SMS reminder (68, 69).

## 9 Policy and advocacy efforts of NCDs in Cambodia

Cambodia’s policy and advocacy efforts regarding NCDs focus on promoting prevention, control, and community engagement in order to address risk factors and strengthen healthcare systems. With support from the World Health Organization (WHO), the Cambodian government developed the *National Strategic Plan for the Prevention and Control of NCDs (2022–30)*, which prioritized reducing tobacco use, unhealthy diets (reducing salt and sugar consumption), physical inactivity, and harmful alcohol consumption (70). This was later expanded into the *Cambodia Multi-Sectoral Action Plan for NCD Prevention and Control (2018–27)*, which emphasizes collaboration across sectors such as health, education, and agriculture to tackle NCDs (71).

Advocacy efforts have been led by organizations such as the Cambodia Movement for Health (CMH) and the NCD Alliance Cambodia, which have pushed for stronger tobacco and alcohol control policies. These include the enforcement of the Tobacco Control Law in 2015 and the implementation of restrictions on alcohol advertising (72). Strengthening the integration of primary healthcare and enhancing multi-sectoral coordination are critical for sustaining Cambodia’s response to NCDs. Creating enabling environments—in homes, villages, cities, and across society—is essential to making healthy choices easier. To achieve this, the multi-sectoral action plan must also aim to break the cycle of poverty and NCDs (71). The country must adopt and promote strong, evidence-based policies and practices for the prevention and control of NCDs. A key to a sustainable solution lies in understanding who is affected and under what circumstances. This understanding should guide advocacy and policymaking that targets individuals, communities, and environmental conditions. The plan must also address the underlying social, environmental, and commercial determinants of health to improve healthcare access and encourage healthier lifestyles—ultimately contributing to sustainable development.

### 9.1 Government initiatives

Cambodia has significantly scaled up its response to NCDs, which now account for 64% of all deaths nationwide (1). In 2022, the Ministry of Health launched the *NCD Benchmarks Framework (2022–30)*, aligning with WHO’s Global Monitoring Framework to reinforce policy implementation and multi-sectoral coordination (70). A major advancement was the nationwide expansion of the HEARTS initiative in 2023, which has trained over 1,200 primary healthcare workers in standardized hypertension and diabetes management across 15 provinces—substantially improving NCD care access in rural areas (73). Additionally, Cambodia strengthened tobacco control in 2023 by banning e-cigarettes and increasing excise taxes, building on the 2015 Tobacco Control Law (40). NCD services are now being integrated into the country’s Universal Health Coverage system, especially through the National Social Security Fund and the Health Equity Fund, to enhance affordability and accessibility for vulnerable populations (37). *The National Action Plan for NCD Prevention and Control (2021–25)* aligns with the WHO Global Action Plan and focuses on health promotion, early detection, and improved access to treatment.

Despite this progress, critical gaps remain. NCD programs receive only 3.2% of total health expenditure, and only 32% of rural health centers currently provide basic NCD services (34). To address dietary risks, the government adopted a *National Salt Reduction Strategy in 2023*, aiming for a 15% reduction in population salt intake by 2025 (7, 34). However, challenges such as urban–rural disparities in healthcare and weak enforcement of alcohol regulations and advertising restrictions still persist. Moving forward, Cambodia must prioritize the expansion of digital health solutions, the strengthening of primary healthcare services, and the adoption of fiscal policies for population-wide impact.

### 9.2 International collaborations

Recognizing the growing burden of NCDs, the Cambodian government has introduced various advocacy and policy initiatives with support from international partners. Organizations such as WHO Cambodia, GIZ Cambodia, PATH, and FHI 360 have contributed to NCD prevention through initiatives such as community-based screenings for diabetes and hypertension, as well as WHO STEPwise surveys (7, 57–60). International collaboration is also vital in the field of research and publication. It enhances the quality of outputs, promotes networking among researchers, facilitates access to funding and technical expertise, and broadens the dissemination of knowledge (74). Like many countries in Southeast Asia, Cambodia is grappling with the growing burden of NCDs. Research that focuses on their prevalence and associated risk factors has been instrumental in advancing the country’s health system and has contributed significantly to regional health progress (9). To raise awareness of these issues and the health consequences of urbanization, public lectures should be organized. These should introduce the topic of NCDs and their risk factors across various disciplines while also enhancing the research skills of young scholars in this field.

## 10 Future directions to address NCDs in Cambodia

**Figure 1:**
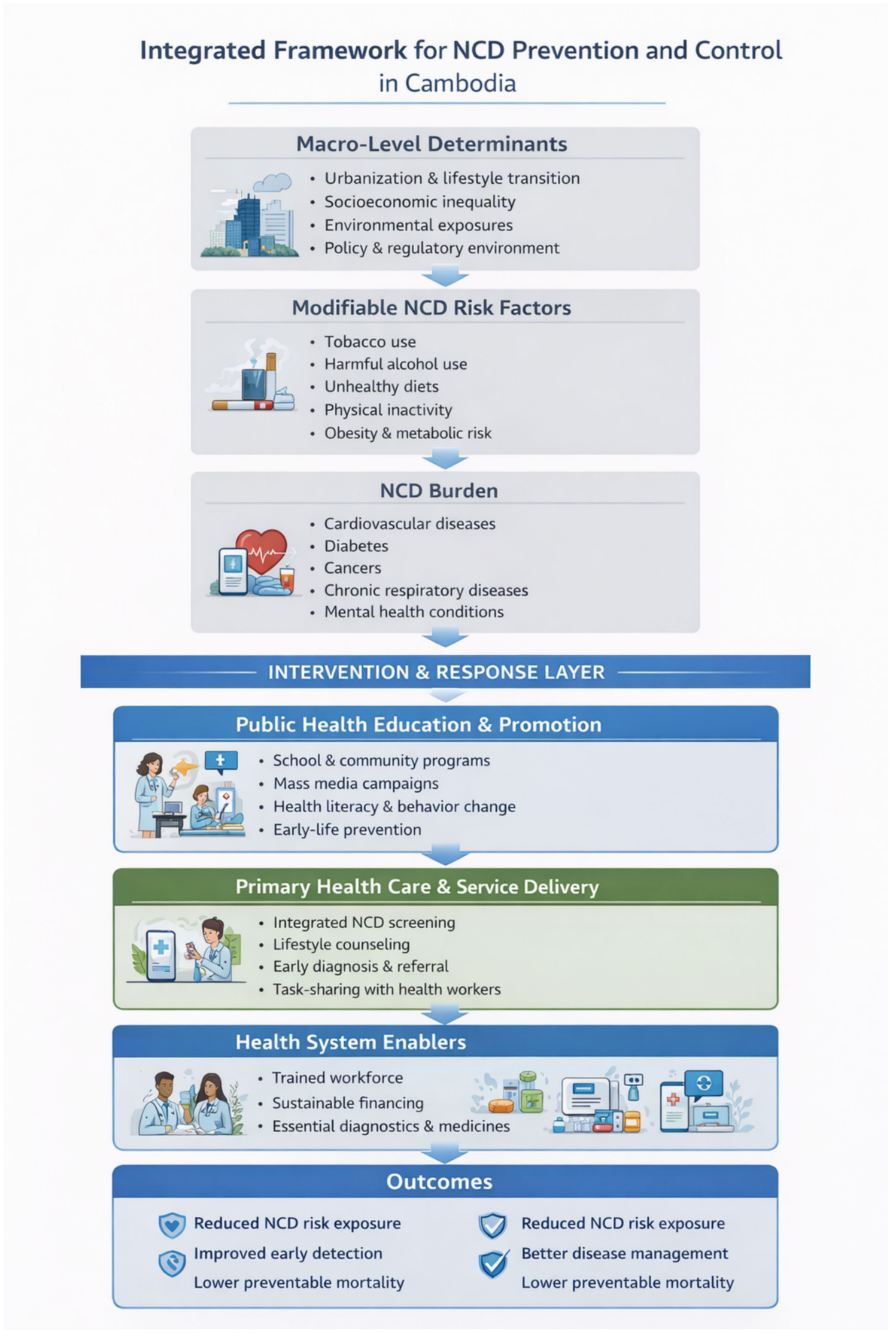
Conceptual framework linking NCD drivers, public health education, and health system responses in Cambodia.

The Cambodian government has implemented a range of programs aimed at addressing the leading causes of premature mortality, with particular emphasis on improving maternal and child health, alongside the rapid scale-up of malaria, tuberculosis, and tetanus control initiatives (75). However, despite their established and growing burden, major NCDs have received comparatively limited attention. Lifestyle-related risk factors—such as poor dietary habits, reduced physical activity, increasing work-related stress, and high rates of tobacco use—have contributed to Cambodia’s ongoing demographic and epidemiological transition (7, 76). Moreover, the high proportion of the population lacking health insurance and exposed to costly medical care highlights how chronic illnesses often lead to extreme poverty. Over time, the absence of a universal healthcare system has resulted in rising healthcare costs associated with chronic disease management, contributing to substantial productivity losses and socioeconomic strain (77). This situation raises critical concerns about the need to prioritize NCDs within the context of regional and international health security. The growing burden on health and welfare systems not only threatens Cambodia’s economic development and stable governance but also undermines the region’s capacity to respond effectively to broader security challenges. To address these concerns, it is essential to collect and analyze robust data on the leading causes of death, key drivers of disability, and specific indicators related to cardiovascular diseases, cancers, chronic respiratory diseases, and diabetes. Such evidence is vital for designing effective, population-based health interventions and services. Addressing the long-term implications of NCDs will be crucial to safeguarding Cambodia’s public health and contributing to a stable global health landscape. Continued multi-sectoral collaboration and sustained advocacy efforts will be essential in reducing the rising burden of NCDs across the country. As climate change is expected to exacerbate NCD risks through increased heat exposure, food insecurity, and worsening air pollution. Future strategies must integrate climate resilience into NCD prevention frameworks. Figure 1 summarizes the conceptual framework linking macro-level determinants, modifiable risk factors, and the NCD burden in Cambodia with public health education, primary healthcare delivery, and key health system enablers. The framework illustrates how education and prevention strategies can mediate risk exposure and support earlier detection and improved long-term management of NCDs. Strengthened health system enablers—including workforce capacity, financing, diagnostics, and digital surveillance—support sustainable NCD prevention and control. While developed in the Cambodian context, the framework is applicable to other LMICs undergoing epidemiological transition.

## 11 Recommendations

This review synthesizes available evidence on the prevalence, trends, and distribution of NCDs in Cambodia, highlighting their growing contribution to morbidity and mortality. NCDs are now responsible for the majority of premature deaths nationwide, with cardiovascular diseases, diabetes, cancers, and chronic respiratory conditions accounting for the largest share of the burden. These conditions are closely associated with modifiable behavioral risk factors, including tobacco use, harmful alcohol consumption, unhealthy diets, physical inactivity, and environmental exposures such as indoor air pollution. Nonetheless, significant gaps remain in the understanding of NCDs in Cambodia. These include a lack of comprehensive data on prevalence and risk factors, limited research on effective intervention strategies, insufficient documentation of long-term management outcomes, weak healthcare infrastructure, low levels of health literacy, and inadequate coordination across sectors.

To effectively tackle Cambodia’s growing NCD burden, the government must adopt a comprehensive, multi-sectoral response that prioritizes prevention, early detection, and equitable access to care. This includes scaling up health promotion initiatives, implementing targeted community interventions, and strengthening primary healthcare services to be more accessible and responsive to local needs—particularly in underserved rural areas.

This review strongly recommends the development of a national NCD strategy that enhances routine screening, improves public awareness and health education, builds health system capacity, and ensures research findings are effectively translated into policy and practice. A robust and sustained national response will require strong political will, cross-sector collaboration, and increased investment in prevention and health promotion.

To translate these priorities into measurable public health gains by 2030, a set of interlinked, education-centered interventions is required. First, community-based NCD screening should be expanded to all 25 provinces, coupled with structured health education and counseling to ensure that early detection leads to informed care-seeking and sustained behavior change. Second, nationwide, school-based nutrition and physical activity programs should be implemented to embed health literacy and healthy behaviors from an early age, thereby addressing NCD risk across the life course. Third, essential NCD prevention and management services should be integrated into at least 80% of rural health centers, with frontline providers trained to deliver lifestyle education, risk communication, and follow-up counseling alongside clinical care.

Adequate and sustainable financing is a critical enabler of effective health promotion. Increasing national funding for NCDs from 3.2% to at least 7% of total health expenditure would better reflect the growing disease burden and support scaled-up prevention, education, and service delivery. In parallel, the establishment of a national digital NCD surveillance platform would strengthen data collection, monitoring, and feedback loops, enabling evidence-based targeting of public health education and promotion strategies. Stronger enforcement of tobacco and alcohol control laws, including routine compliance audits, is also essential to reduce population exposure to major behavioral risk factors and to reinforce health messaging through regulatory action.

To maximize population reach and improve health literacy at scale, innovative delivery approaches to health education should be prioritized. SMS-based telehealth platforms offer a low-cost and highly scalable mechanism for disseminating public health information. The existing experience of the Telecommunication Regulator of Cambodia in delivering SMS reminders for communicable diseases, including HIV and AIDS, demonstrates the feasibility of this approach. Expanding this platform to include NCD-related education—such as guidance on nutrition, physical activity, symptom recognition, and treatment adherence—would provide an efficient means of strengthening population-wide health awareness. Collectively, these interventions constitute a realistic and integrated roadmap for reducing the NCD burden in Cambodia while promoting long-term population health and economic sustainability through strengthened public health education and prevention.

## 12 Limitations and priority areas for future research

NCD research in Cambodia is constrained by limited longitudinal data, inconsistent surveillance, and a lack of rigorous evaluations of health education and promotion interventions. Future research should prioritize assessing the effectiveness of community-based education programs, digital health tools for behavior change, and models for integrating prevention into primary care.

## 13 Conclusion

In conclusion, NCDs now constitute the dominant health challenge in Cambodia, reflecting a rapid epidemiological transition common to many LMICs. Limited research and inadequate data continue to hinder a comprehensive understanding of their prevalence and associated risk factors. Key drivers of the rise in NCDs include alcohol consumption, tobacco use, unhealthy diets, and physical inactivity. Cambodia must strengthen data collection systems, develop evidence-based policies, and implement targeted health initiatives to effectively tackle this challenge. Promoting healthy lifestyles, raising public awareness, and ensuring equitable access to healthcare services are critical steps toward reducing the impact of NCDs. Furthermore, leveraging external financial and technological support is essential to bolster domestic capacity. Addressing NCDs requires coordinated efforts among all stakeholders and a robust multi-sectoral approach. This review contributes to a broader understanding of NCDs in Cambodia by outlining current knowledge, identifying key risk factors, and emphasizing the need for focused interventions. With sustained attention and investment, Cambodia has the potential to significantly enhance the health and well-being of its population. Without urgent and coordinated action, NCDs could cause a loss of up to 6.6% of Cambodia’s GDP by 2030. Prioritizing NCD prevention and control is therefore not only a health priority but also a critical step toward achieving Cambodia’s UHC and SDG and Cambodian pentagonal strategies.

## Data Availability

The original contributions presented in the study are included in the article, further inquiries can be directed to the corresponding author.
